# Lung cancer risk test trial: study design, participant baseline characteristics, bronchoscopy safety, and establishment of a biospecimen repository

**DOI:** 10.1186/s12890-016-0178-4

**Published:** 2016-01-22

**Authors:** E. L. Crawford, A. Levin, F. Safi, M. Lu, A. Baugh, X. Zhang, J. Yeo, S. A. Khuder, A. M. Boulos, P. Nana-Sinkam, P. P. Massion, D. A. Arenberg, D. Midthun, P. J. Mazzone, S. D. Nathan, R. Wainz, G. Silvestri, J. Tita, J. C. Willey

**Affiliations:** Department of Pulmonary and Critical Care, The University of Toledo Medical Center, Toledo, OH USA; Department of Biostatistics, Henry Ford Hospital System, Detroit, MI USA; Ohio State University James Comprehensive Cancer Center and Solove Research Institute, Columbus, OH USA; Thoracic Program, Vanderbilt Ingram Cancer Center, Nashville, TN USA; University of Michigan, Ann Arbor, MI USA; Mayo Clinic, Rochester, MN USA; Cleveland Clinic, Cleveland, OH USA; Inova Fairfax Hospital, Falls Church, VA USA; The Toledo Hospital, Toledo, OH USA; Medical University of South Carolina, Charleston, SC USA; Mercy/St. Vincent’s Hospital, Toledo, OH USA

**Keywords:** Lung cancer risk test, Hereditary lung cancer risk, Normal bronchial epithelial cells, Lung cancer screening, Bronchoscopy safety, Bronchial brush safety

## Abstract

**Background:**

The Lung Cancer Risk Test (LCRT) trial is a prospective cohort study comparing lung cancer incidence among persons with a positive or negative value for the LCRT, a 15 gene test measured in normal bronchial epithelial cells (NBEC). The purpose of this article is to describe the study design, primary endpoint, and safety; baseline characteristics of enrolled individuals; and establishment of a bio-specimen repository.

**Methods/Design:**

Eligible participants were aged 50–90 years, current or former smokers with 20 pack-years or more cigarette smoking history, free of lung cancer, and willing to undergo bronchoscopic brush biopsy for NBEC sample collection. NBEC, peripheral blood samples, baseline CT, and medical and demographic data were collected from each subject.

**Discussion:**

Over a two-year span (2010–2012), 403 subjects were enrolled at 12 sites. At baseline 384 subjects remained in study and mean age and smoking history were 62.9 years and 50.4 pack-years respectively, with 34 % current smokers. Obstructive lung disease (FEV1/FVC <0.7) was present in 157 (54 %). No severe adverse events were associated with bronchoscopic brushing. An NBEC and matched peripheral blood bio-specimen repository was established.

The demographic composition of the enrolled group is representative of the population for which the LCRT is intended. Specifically, based on baseline population characteristics we expect lung cancer incidence in this cohort to be representative of the population eligible for low-dose Computed Tomography (LDCT) lung cancer screening. Collection of NBEC by bronchial brush biopsy/bronchoscopy was safe and well-tolerated in this population. These findings support the feasibility of testing LCRT clinical utility in this prospective study. If validated, the LCRT has the potential to significantly narrow the population of individuals requiring annual low-dose helical CT screening for early detection of lung cancer and delay the onset of screening for individuals with results indicating low lung cancer risk. For these individuals, the small risk incurred by undergoing once in a lifetime bronchoscopic sample collection for LCRT may be offset by a reduction in their CT-related risks. The LCRT biospecimen repository will enable additional studies of genetic basis for COPD and/or lung cancer risk.

**Trial registration:**

The LCRT Study, NCT 01130285, was registered with Clinicaltrials.gov on May 24, 2010.

**Electronic supplementary material:**

The online version of this article (doi:10.1186/s12890-016-0178-4) contains supplementary material, which is available to authorized users.

## Background

Lung cancer claimed nearly 160,000 lives in 2014 in the United States alone [1]. Prevention efforts have reduced cigarette smoking prevalence from about 50 % in 1960 to less than 20 % today but, due to past and continued cigarette smoking and the lack of effective treatment for advanced disease, lung cancer kills more than the next three most deadly cancers (breast, colon, prostate) combined and is expected to do so for decades to come [1]. Because prognosis is related to stage, there has long been interest in detecting lung cancer in early stage when it is amenable to potentially curative treatment. Thus, it is notable that the US Preventive Services Task Force (USPSTF) now recommends lung cancer screening with LDCT for healthy individuals at high risk for lung cancer on the basis of evidence that it will detect the majority of lung cancers in early stage and thereby reduce lung cancer mortality by > 20 % [2, 3]. However, the overall benefit of screening is associated with adverse consequences, including identification of large numbers of nodules, most of which will be nonmalignant, and the complications, costs, and anxiety associated with diagnostic tests [4]. These adverse consequences could be reduced by restricting screening eligibility to only those at greatest risk. Among the approximately 8 million subjects eligible for screening according to current criteria, which include smoking history ≥30 pack-years and age 55–80 years [3, 5], risk varies widely from less than 0.08 % per year to over 1 % per year [6–13]. As such, a large majority of screened individuals will not develop lung cancer in their lifetime and the overall benefit of screening is reduced by the adverse events and large cost associated with screening subjects who will not benefit due to low risk. For these reasons, there is increasing interest in the development of an accurate diagnostic molecular test for lung cancer risk that will more accurately stratify subjects for screening. It is expected that limiting screening to those with a positive risk test will reduce the high cost and side effects of screening programs.

Different approaches are currently in progress to develop a molecular diagnostic test for lung cancer risk in the group eligible for annual CT screening based on demographic criteria. These approaches may be divided into two broad categories, early diagnosis and hereditary risk.

The early diagnosis strategy is to detect lung cancers in early stage before symptoms occur so that they can be treated with high chance for cure. This category includes approaches to identify pre-clinical early lung cancer based on blood tests for circulating proteins, antibodies, and/or microRNA [14–21], or gene expression tests measured in non-cancer bronchial or nasal airway epithelium that reflect presence of lung cancer due to a field effect [22–24]. Because these tests are for early detection they will need to be repeated periodically. A positive test will inform a decision regarding more conservative or more rigorous assessment for presence of lung cancer, including chest CT and/or PET-CT, followed by biopsy. If the intended use is to serve as the primary screening method, an early diagnosis test will need to demonstrate non-inferiority relative to the screening test currently recommended by the USPSTF, annual low dose helical CT.

The hereditary risk test strategy is to identify individuals who have a genetic predisposition to lung cancer so that they can be prioritized for annual chest CT screening. Approaches to identify hereditary risk include a) genome wide association studies (GWAS) to discover DNA polymorphisms associated with lung cancer [25, 26] and b) studies to identify risk-associated proximate phenotypic markers [5]. The Lung Cancer Risk Test (LCRT) falls into this latter category. The LCRT is a 15 gene test measured in grossly normal bronchial epithelial cells (NBEC) obtained through bronchial brush biopsy [5]. The proximate phenotypic markers of hereditary risk comprised by the LCRT are key protective antioxidant, DNA repair, and cell cycle control genes that are sub-optimally regulated in normal bronchial epithelial cells (NBEC). The rationale for this approach is that sub-optimal NBEC regulation of a protective gene has greater effect on risk than an individual single nucleotide polymorphism (SNP). This conclusion is based on results of previous studies in which we identified *cis*-regulatory SNPs associated with sub-optimal regulation of genes comprised by the LCRT, including ERCC5 [27]; [Zhang, submitted] and CEBPG [28]. For example, we identified two *cis*-regulatory SNPs that independently contribute to regulation of ERCC5 transcript abundance [27]; [Zhang, submitted]. Thus, a proximate phenotype based on sub-optimal NBEC regulation of a protective gene enriches for *cis*-regulatory SNPs that may contribute to risk.

The clinical setting for LCRT biomarker intended use is individuals who are approaching annual CT screening eligibility according to USPSTF criteria [2]. In order to have clinical utility it is important that the test be both accurate and safe to perform in this intended population. In an effort to assess the accuracy and safety of the LCRT we initiated a multi-site prospective cohort trial. The purpose of this report is to describe 1) the LCRT trial study design and primary endpoint, 2) baseline characteristics of enrolled individuals including demographic and lung function data, and 3) secondary endpoints reached thus far, including a) analysis of safety for the bronchoscopic brush method used to obtain samples for LCRT testing, and b) establishment of a biospecimen repository containing NBEC and peripheral blood samples collected from the LCRT cohort.

## Methods

### Study design

This LCRT study (Clinicaltrials.gov, NCT 01130285) was conducted after approval by an institutional review board at each participating institution (University of Toledo Medical Center, Mayo Clinic, University of Michigan, The Toledo Hospital, Ohio State University, Vanderbilt University Medical Center/Tennessee Valley VA Medical Center, Henry Ford Health System, National Jewish Health, Medical University of South Carolina, Inova Fairfax Hospital, Cleveland Clinic Foundation and Mercy St. Vincent Medical Center, see Additional file 1: Table S1) and under a Federal Drug Administration (FDA) approved Investigational Device Exemption (IDE G090273). The original design to assess the clinical utility of the LCRT biomarker was a prospective, blinded, nested case–control study. The original primary endpoint was prediction of risk for development of lung cancer with an odds ratio of at least 5.0. It was estimated that there would have been sufficient power to test this endpoint by enrolling approximately 800 subjects and following them for 3 years, resulting in identification of at least 15 prospective lung cancer cases. LCRT analysis would then be conducted in NBEC of the 15 cases and 120 matched controls. However, the study was revised to a prospective cohort design due to a) advances in technology that enable cost-effective measurement of LCRT in all subjects, and b) the greater power associated with this design. The new design and primary endpoints are described below.

The secondary endpoints and analyses are unchanged and include: 1) determination of study safety at day 30, 2) establishment and maintenance of a biospecimen repository of biological specimens derived from NBEC [RNA and cytology slides] and corresponding blood samples [peripheral blood leukocyte Buffy Coat and frozen plasma] from the subjects enrolled, 3) analysis of the predictive ability of LCRT positive for lung cancer including sensitivity, specificity, positive predictive value, and negative predictive value, 4) calculation of absolute risk of LCRT positive for lung cancer and, 5) measurement of the incidence of lung cancer in the study cohort every two years until the end of study. Additionally, we will explore the influence of demographic or clinical variables for lung cancer on the predictive ability of LCRT.

### Revised study design

After development of a novel targeted NGS platform [29], we implemented LCRT measurement on this platform. The higher throughput of the NGS method enables cost-effective analysis of samples from all 384 subjects and conversion to a prospective cohort study with greater power compared to the original nested case–control design. We plan to assess association of the LCRT value with development of lung cancer in this cohort through follow-up every one to two years for up to 20 years. We will estimate disease-free probabilities for different measured LCRT values at six and eight years of follow up. The primary endpoint will be the prediction of risk for development of lung cancer with a risk ratio of at least 5.0 and we expect to reach this endpoint at the six year follow-up.

Assuming a 20 % rate of failure to re-contact (due to death or other factors), approximately 300 individuals from the cohort will be available for analysis. Based on the demographic characteristics of the LCRT cohort, the expected cumulative incidence at six years following enrollment (which will be reached for all subjects between 2016 and 2018) is >5 %. Assuming a two-tailed test of significance and a type-1 error rate of 0.05, there will be >80 % power to detect a risk ratio associated with LCRT positivity of ≥ 2.45, 1.82, 1.65, 1.57, 1.49, and 1.42 for cumulative incidence rates of lung cancer of 1 %, 2 %, 3 %, 4 %, 5 %, and 6 %, respectively, in the cohort at the six year follow-up. Thus, this proposed study is more than adequately powered to detect even modest LCRT effects at the next planned follow-up. In addition to the risk ratio associated with a positive LCRT, we will also calculate the concordance index of the test based on the estimated Cox proportional hazards model. The concordance index in the Cox model is the correlate to the area under the receiver operator characteristic curve for a logistic regression model. We will use it to measure LCRT biomarker accuracy in the full cohort analysis.

### Participants

To participate in the study, subjects had to be willing and able to provide and sign both written Informed Consent and Health Insurance Portability and Accountability Act Authorization (HIPAA) forms for this study, undergo bronchoscopy and phlebotomy procedures for the collection of biological specimens and follow up interviews and CT scans. Entry criteria required subjects to be at high demographic risk for lung cancer based on age 50–90 years, and a minimum of 20 pack-years of cigarette smoking history, but to have low likelihood for lung cancer at the time of bronchoscopy. Both current (defined as self-reported regular use of cigarettes) and former cigarette smokers were eligible. Consent included bronchial brush biopsy to obtain NBEC samples at time of either a) standard of care (SOC) bronchoscopy for a clinical indication for bronchoscopy, b) a study-driven (SD) bronchoscopy, or c) bronchoscopy done for another research study to which they had consented (also considered to be SD). Subjects had to be without a diagnosis of lung cancer prior to or at enrollment. Women with the potential for pregnancy had to have a negative result on a pregnancy test. Subjects were excluded if they were previously diagnosed or treated for lung cancer or had a high pretest likelihood of lung cancer, if they were positive for hepatitis B, C, HIV, or had active TB or if the physician deemed them to be medically inappropriate due to safety concerns. Also excluded were children, pregnant women, prisoners, mentally disabled, those that had received a double lung transplantation, radiation or chemotherapy of any kind within the last month and those scheduled to receive either radiation or chemotherapy.

### Recruitment strategies

Twelve medical institutions participated in the LCRT (Clinicaltrials.gov, NCT 01130285, Additional file 1: Table S1).

Participants were recruited through physician referral as well as by advertisements in local newspapers, on institutional web sites and through Clinical Trial.gov. The goal was to enroll a sample representative of the U.S. population at high risk of lung cancer death based on demographic criteria.

### Enrollment

Subjects were considered enrolled in the LCRT study when they underwent the study procedure (bronchial brush biopsy with NBEC sample collection). All enrolled subjects had a CT of the chest performed within 3 months prior to study entry or a research driven CT scan within two weeks after study entry to rule out prevalent lung cancer. Study eligibility, including smoking history, was assessed through initial contact interview by a trained clinical coordinator at each site. The initial Contact Report Form (CRF) was designed to allow for computation of number of pack-years of cigarettes smoked as well as a detailed smoking history that included information on periods of smoking cessation and use of other forms of tobacco such as pipes and cigars. The CRF also contained questions on personal history of selected diseases, stroke, and diabetes, family history of lung cancer, occupational history (jobs and industries either previously demonstrated or thought to be associated with increased risk for lung disease or lung cancer), education, and marital status.

### Sample collection

Standardized sample collection kits were provided to each site. Kits contained supplies for the collection and labeling of biological samples including a disposable bronchial cytology brush (ConMed Corporation, Utica, NY ref.#149) for the collection of NBEC, a 10 ml K_2_-EDTA vacutainer tube (Becton, Dickinson and Company, Franklin Lakes, NJ ref.#366643) for the collection of whole blood and barcoded stickers. Following positioning of the bronchoscope, the cytology brush was inserted and NBEC were collected from a grossly normal region of either main stem bronchus. For SOC bronchoscopies, this occurred immediately after the diagnostic procedures on the opposite side or in a separate area from the lung region under clinical investigation. If the patient had received a lung transplant, the specimen was obtained from the recipient native mainstem bronchus. The brush was withdrawn, shaken into a tube of normal saline chilled on ice and re-inserted into the bronchoscope for collection of additional NBEC. This procedure was repeated a total of 5–10 times. After the last brushing, the cytology brush was shaken in the saline and then dabbed onto a glass slide to enable assessment by a pathologist. Immediately prior to or immediately following bronchoscopy, approximately 10 ml of whole blood was obtained using standard phlebotomy techniques into a K_2_-EDTA vacutainer tube. Blood and NBEC samples were transferred to the lab within 10 min. for processing and stabilization, which was initiated within 1 hour post-collection.

### Follow up

Subjects enrolled into the study were followed at 30 days for adverse events (AE) and serious adverse events (SAE) possibly related to the study procedure and then every 3 months throughout the first two years following enrollment. A research driven CT was done at the one and two year anniversaries of enrollment if a standard of care CT was not done within three months of the anniversary. The next follow-up is planned for 2016 with another in 2018. At each follow-up subjects will receive medical record review and phone interview. Those who meet USPSTF guidelines will be encouraged to enter the closest CT screening program for early detection of lung cancer. Those who do not meet current reimbursement criteria for CT screening will receive a study driven chest CT.

### Safety analysis: adverse events and serious adverse events

Subjects were monitored for all adverse events (AE) immediately following bronchoscopy until deemed medically stable and ready for discharge and again at 30 days after study enrollment by way of a phone call with the subject. Subjects were monitored for serious adverse events (SAE) for two years following enrollment.

Possible AEs included, but were not limited to, fatigue, muscle aches, bitter taste in mouth, dry or sore throat, hoarseness, fever [greater than 100 °F for more than 24 hours], bronchospasm, arrhythmia, pneumothorax, hemoptysis, shortness of breath and infections. A SAE was defined as any serious effect on the health or safety or any life-threatening problem or death caused by, or associated with the study procedure if that effect, problem or death was not previously identified in the investigational plan or application. These included hospitalization [≥24 hours], death, disability, or any event that require intervention to prevent damage.

AEs and SAEs were documented and classified in terms of severity [mild, moderate, severe], expectedness [expected or unexpected] and relatedness [unlikely, possibly, probably or unknown]. A medical monitor at the data coordinating center (Dr. Paul Kvale at Henry Ford Health System) worked closely with each site PI and ultimately was responsible for the final determination of SAE relatedness. Treatments or interventions and outcomes also were documented.

### Statistical analysis

Statistical significance was determined using an F-test of equality of variances following by a Student’s t-test for comparison of groups on continuous variables and Chi square or Fisher exact test for categorical variables. Differences were considered significant if *p* < 0.05. Power analysis was conducted as described above in the Revised Study Design section.

## Results

Here we present the baseline characteristics of the enrolled LCRT cohort, and results for secondary endpoints that have been reached including safety analysis and establishment of the NBEC and peripheral blood sample biospecimen repository.

### Enrollment

**Fig. 1:**
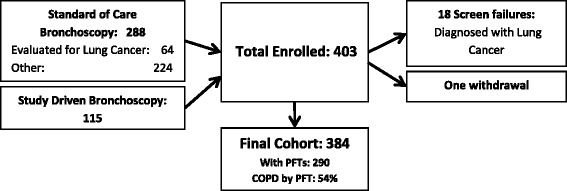
Summary of Enrolled Cohort

Accrual for the LCRT study was completed in March 2012. We enrolled 403 subjects with demographic risk factors for lung cancer into a prospective multi-site, blinded LCRT study, performed bronchoscopy at enrollment, and collected NBEC and blood (buffy coat and plasma) samples from each subject (Fig. 1). Of the 403 subjects enrolled, 288 were enrolled at the time of a standard of care (SOC) bronchoscopy done for diagnostic purposes and 115 were enrolled at time of a volunteer study driven (SD) bronchoscopy. Of the 288 SOC bronchoscopies, 64 were done to evaluate for lung cancer, 34 for monitoring following lung transplantation, and the remaining 190 for a variety of indications. Of the 403 subjects enrolled, 18 were removed from the study as screen failures due to diagnosis of prevalent lung cancer at enrolling bronchoscopy or subsequent tests and one subject withdrew from the study leaving 384 subjects in the cohort. We conducted a descriptive analysis of baseline data for the 384 remaining subjects.

### Demographic information

Subject population characteristics are shown in Table 1. Of the 384 subjects, mean age was 62.9 ± 8.2 years with a mean smoking history of 50.4 pack years. Thirty-four percent were current smokers and approximately 10 % were concomitant cigar and/or pipe smokers. The cohort included 213 males (55 %) and 171 females (45 %), 89 % Caucasians, 10 % African Americans, and 1 % other. Sixty percent of subjects were married or living with a partner, 30 % were widowed, and 10 % were single. A majority (66 %) were high school graduates with or without some college less than a bachelor’s degree, 10 % held a bachelor’s degree and 6 % held an advanced degree. Reported income was less than $40,000 per year pt?>in 37 % of subjects although 31 % of subjects (120 individuals) chose not to provide household income information. Forty-six percent were retired and 17 % were disabled (Table 1). Work-related exposures were reported by 234 (61 %) of subjects with the highest percentages being asbestos (*n* = 54, 14 %), farming (*n* = 41, 11 %), chemicals or plastics (10 %), welding (10 %), foundry or steel milling (9 %), and painting (9 %) (Additional file 2: Table S2). Each subject had a chest CT scan at the time of enrollment; 242 subjects (63 %) had a clinically indicated (standard of care) CT scan within three months prior to enrollment and the remaining 142 (37 %) had a research driven CT scan within 2 weeks of enrollment. Twelve percent of subjects were undergoing evaluation for lung cancer at time of enrollment and were negative for cancer (Additional file 3: Table S3). Based on responses to baseline questionnaire, self-reported prevalence of chronic obstructive pulmonary disease (COPD) was 41 % (*n* = 156), chronic bronchitis 18 % (*n* = 68), and emphysema 28 % (*n* = 106) (Additional file 3: Table S3). Because Pulmonary Function Test (PFT) data were available for most subjects, it was possible to compare self-reported COPD prevalence to test data (see below). Prevalence of other self-reported lung diseases were: interstitial lung disease 9 % (*n* = 35), and sarcoidosis 3 % (*n* = 10) (Additional file 3: Table S3).

**Table 1:** LCRT Subject Characteristics

Baseline characteristics	*n* = 384
Age in years [mean (SD^a^)]	62.9 (8.2)
Age in years [median]	62
Male	213 (55 %)
Female	171 (45 %)
Caucasian	343 (89 %)
African American	37 (10 %)
Other or not reported	4 (1 %)
Cigarette pack years [mean (SD)]	50.4 (25.5)
Cigarette pack years [median]	43
Age in years at smoking inception [mean (SD)]	16.1, 3.8
Age in years at smoking inception [median]	16
Total years of smoking [mean (SD)]	37.4 (10)
Total years of smoking [median]	38
History of cigar use	35 (9 %)
History of pipe use	29 (8 %)
Married or living as married	231 (60 %)
Widowed	116 (30 %)
Single	37 (10 %)
Less than high school education	52 (14 %)
High school diploma or GED^b^	118 (31 %)
Associate degree or some college	136 (35 %)
Bachelor's degree	40 (10 %)
Graduate degree	24 (6 %)
Other or not reported	14 (4 %)
Employed	109 (28 %)
Unemployed	30 (8 %)
Retired	175 (46 %)
Disabled	64 (17 %)
Other or not reported	6 (2 %)
Income < $40,000/year	141 (37 %)
Income > $40,000/year	123 (32 %)
Other or not reported	120 (31 %)

^a^SD = standard deviation

^b^GED = Graduate Educational Development

### SOC vs SD bronchoscopy characteristics

The intended population for the LCRT includes both subjects for whom diagnostic bronchoscopy is indicated who also will benefit from LCRT measurement and subjects who will have bronchoscopy only to obtain NBEC samples for LCRT measurement. Therefore, we compared baseline characteristics between the SOC and SD bronchoscopy subject groups, which represent each of these respective intended population categories. Of 384 subjects enrolled, bronchoscopy was SOC in 269 (70 %) and SD in 115 (30 %). There were no significant differences in in pack years smoked (Additional file 4: Table S4). SD subjects were slightly younger (mean age of 61.5 compared to 63.6, *p* = 0.021), more likely to be current smokers (55 % vs. 25 %, *p* < 0.001), and less likely to have COPD (41 % vs. 60 %, *p* = 0.002) (Additional file 4: Table S4).

### Lung cancer screening eligible sub-group

The USPSTF age and smoking pack year eligibility criteria for lung cancer screening by annual low-dose helical chest CT are 55–80 years and a minimum of 30 pack-years, respectively. Among subjects enrolled into the LCRT study, 253/384 (65.9 %) were eligible for annual screening at enrollment, according to these criteria. Seventy subjects did not meet the minimum age criterion at time of enrollment. By the 2016 follow up time point, 45 of these 70 will be eligible for screening and 69/70 will be eligible by the 2018 follow up.

### Chronic obstructive pulmonary disease

We assessed COPD status in the enrolled cohort because COPD is an independent risk factor for lung cancer [30–38]. COPD was defined using GOLD criteria based on pulmonary function test (PFT) data [39]. Demographic information relative to COPD status is displayed in Table 2. PFT information was available for 290 subjects. Fifty-four percent of these (157 subjects) had COPD based on PFT. Among the 157 subjects with COPD based on PFT, COPD severity was GOLD stage 2 or worse in more than 70 % based on established criteria [39]. Mean FEV1/FVC was 0.52 for the 157 subjects with COPD (all stages) compared to 0.78 for the 133 without COPD. Those with COPD were more likely to be male (62 % vs. 38 % female, *p* = 0.027) and have a higher mean pack year smoking history (56 vs. 45 for non-COPD, *p* < 0.001). No differences were noted in age, race or smoking status (current vs. former smokers) (Table 2.)

**Table 2:** Chronic Obstructive Pulmonary Disease by PFT

Classification	n	M/F^a^	Mean age	Race	Smoking status	Pack years	FEV1%^d^	FEV1/FVC^e^
			in years	C/AA/Other^b^	current/former	smoked^c^		
No COPD	133	65 / 68	62	113 / 16 / 4	46 / 87	45	80	0.78
COPD (all)	157	97 / 60	63	145 / 12 / 0	50 / 107	56	58	0.52
COPD (stage 1)	45	32 / 12	62	41 / 4 / 0	22 / 23	54	76	0.59
COPD (stage 2)	77	48 / 29	64	73 / 4 / 0	22 / 55	57	57	0.55
COPD (stage 3)	26	15 / 11	64	23 / 3 / 0	5 / 21	53	35	0.39
COPD (stage 4)	7	2 / 5	63	7 / 0 / 0	1 / 6	65	22	0.27
COPD (stage unknown)	2	1 / 1	66	1 / 1 / 0	0 / 2	66	-	0.57
Unknown	94	50 / 44	63	85 / 9 / 0	35 / 6 0	50	-	-

^a^M = male, F = female

^b^C = Caucasian, AA = African-American, Other = other race or race not reported

^c^Pack years = packs of cigarettes smoked per day x years of smoking

^d^FEV1% = forced expiratory volume in 1 second, percent of expected

^e^FEV1/FVC = FEV1/Forced Vital Capacity

Of the 157 subjects with COPD based on PFT criteria, clinical history of COPD based on self-report or chart review was available for 150. Overall, self-reported status matched the diagnosis by PFT in 67 % (Additional file 5: Table S5.).

### Lung transplant

Nine percent (34 subjects) of our cohort had received a (single) lung transplant prior to enrollment. We evaluated differences between lung transplant and non-lung transplant subjects to determine if there were comparable demographic risk factors for lung cancer. Age (62.9 vs. 63.9 years, *p* = 0.202), gender, race and smoking history (51.7 vs. 50.0 pack years, *p* = 0.681) were statistically similar, but 100 % of transplant subjects were former smokers compared to only 63 % of non-transplant subjects (*p* < 0.001). Prevalence of COPD was comparable, 62 % vs. 53 %, *p* = 0.648. Interstitial lung disease, however, was more prevalent among transplant subjects 29 % vs. 7 %, *p* < 0.001 (Additional file 6: Table S6).

### Adverse events

Serious Adverse Events (SAEs) included any serious effects on the health or safety or any life-threatening problems or death caused by, or associated with the study procedures. There were no SAEs attributable to this study for either the 241 SOC bronchoscopy subjects or 142 SD bronchoscopy subjects. Adverse Events (AEs) were collected immediately post-procedure and again at the 30 day follow up. AEs classified as possibly or probably attributable to the study were those associated with bronchoscopy and bronchial brush biopsy such as sore throat, hoarseness, cough, throat swelling, chest soreness, bleeding, fever, fatigue and upper respiratory infection. Since the SOC group received the bronchoscopy as part of their standard-of-care, study related AEs were those associated with the bronchial brushing only. There were no AEs classified as study related among the SOC group.

Among the SD group, there were 11 AEs noted in 9 subjects that were possibly (*n* = 9) or probably (*n* = 2) attributable to study procedures. Additionally, AEs were documented in two additional subjects that were deemed unlikely to be related (Table 3). All AEs were classified as mild.

**Table 3:** Adverse Events (AE)

Subject #	AE Description	Severity	Relatedness	Treatment Notes
1035	Felt poorly (like he had a fever) for 3 days	Mild	Possible	Did not seek treatment or notify study personnel until 30 day follow-up
1044	Upper respiratory tract infection	Mild	Possible	Treated with antibiotics and steroids, infection resolved
1048	Hoarseness for 2 days	Mild	Probable	
1050	Bruising around eyes	Mild	Unlikely	
1057	Bleeding from ears post bronchoscopy	Mild	Possible	
1057	Petechiae around eyes	Mild	Possible	
1059	Cough	Mild	Possible	
1059	Difficulty swallowing	Mild	Possible	
1060	Felt soreness in lung	Mild	Possible	
1061	Dry scratchy area in throat, feels need to cough	Mild	Possible	
1076	Slight cough	Mild	Possible	
1077	Back of throat swollen	Mild	Probable	
1078	Cough	Mild	Unlikely	

### Establishment of NBEC and peripheral blood sample biospecimen repository

Matched blood and NBEC were collected for 361/384 (94 %) subjects and banked in multiple aliquots. Blood samples were processed at each site at the time of collection to generate 2 aliquots of buffy coat and 2–5 aliquots of plasma from each subject. These aliquots were frozen and stored at −80 °C until shipment to the Early Detection Research Network (EDRN) Biorepository in Fredrick, MD. One aliquot of buffy coat was transferred to the University of Toledo for analysis and the other remains in storage. NBEC were stabilized at each site in RNA Later (Ambion, Austin, TX) and shipped along with matching slides to ResearchDx, Irvine, CA. RNA was extracted from NBEC within 24–48 hours of receipt, assessed for quality and quantity and stored in aliquots at −80 °C. One NBEC RNA aliquot was shipped to the University of Toledo for analysis for those samples with a minimum yield of 1 microgram and aliquots for each subject remain in storage at ResearchDx.

At the University of Toledo, genomic DNA (gDNA) was extracted from one aliquot of buffy coat derived from the blood sample from approximately 80 % of subjects and 100 % of these yielded gDNA of sufficient quality and quantity for proposed molecular studies. The quality and quantity of NBEC RNA from approximately 40 % of subjects has been assessed to date. RNA from each sample was treated with DNase I, tested via PCR to ensure removal of contaminating gDNA from the RNA and then reverse transcribed into cDNA. For 90 % of subjects the cDNA generated from these purified NBEC RNA samples was PCR amplifiable and of sufficient quantity to perform LCRT testing. Additional aliquots of RNA remain for the roughly 10 % of samples that did not pass this quality control. Samples from over 120 subjects were used successfully in preliminary targeted next generation sequencing (NGS) RNA sequencing analysis studies.

### Lung cancer incidence

Two years following initiation of the study, 5 subjects (1.3 %) without prevalent lung cancer developed bronchogenic carcinoma. Due to the blinded status of the LCRT study, no further details are available regarding these subjects.

## Discussion

### Enrolled cohort is representative of LCRT target population

The target population of the LCRT biomarker is individuals who meet USPSTF eligibility criteria for annual low dose helical CT screening [2]. The enrollment criteria for the LCRT study included both current and former smokers, individuals with and without concurrent pulmonary disease and/or respiratory exposures as well as both subjects undergoing medically recommended bronchoscopy (SOC group) and volunteers (SD group). At the time of enrollment into the LCRT study, most subjects (66 %) met USPSTF age and smoking pack-year eligibility criteria (55–80 years of age, ≥ 30 pack years). Additionally, most of those not eligible at enrollment will be eligible for screening by the 2016 follow up time point due to increased age, and this fraction is expected to further increase at the 2018 follow up. Therefore, this group is highly representative of the LCRT biomarker target population.

### Feasibility to reach LCRT study endpoint based on cohort characteristics

Based on demographic characteristics of the enrolled population (Table 1), we expect lung cancer incidence in the LCRT study to be similar to the 3.1 % incidence over 3.9 years reported by Bach et al. [40] in which mean age was 60.1 and smoking history of 52 pack-years. The five incidental lung cancers observed two years after initiation of the study are consistent with this rate. Taking into account that some of the 384 study subjects will have died from causes other than bronchogenic carcinoma prior to these time points and that some will be lost to follow up we estimated incidental lung cancers in the cohort based on 300 subjects. As such we expect to observe approximately 12 incidental lung cancers by the 2016 follow up (mean time since enrollment approximately 5 years) and 17 by the 2018 follow up point (mean time since enrollment approximately 7 years), which will be more than sufficient to reach the proposed endpoint of a risk ratio of ≥ 5.0.

### Feasibility of LCRT implementation (safety and acceptance by subjects)

The LCRT biomarker requires a one-time acquisition of NBEC through bronchial brush biopsy at the time of bronchoscopy. In addition to the LCRT study, Department of Defense Lung Cancer Research Program, and NIH recently funded other large studies assessing utility of biomarkers measured in NBEC obtained at bronchoscopy intended to more accurately determine lung cancer risk and/or to enable early lung cancer diagnosis (Massion, Clinicaltrials.gov NCT01475500 CA152662 and CA102353; Spira, Clinicaltrials.gov NCT02504697 DECAMP-2 and CA164783-04; Dubinett, CA152751-05S2). Therefore, it is important to carefully evaluate the safety and comfort of this procedure, which will impact general acceptance by patients and clinicians. Based on published studies, bronchoscopy with or without biopsy is considered a safe procedure and it is used not only for medical purposes but also to conduct research [5, 41–49]. Reported complication rates (also known as serious adverse event/SAE rates) for all bronchoscopy procedures range from 0.08-1.93 % and mortality rates range from 0.004-0.045 % [50–52]. One large Japanese study of almost 50,000 patients who underwent bronchoscopy with brush biopsy in either central or peripheral airways reported a complication (SAE) rate of 0.46 %. This risk of complication is similar to the 0.28-0.32 % complication (SAE) rate reported for colonoscopy [53, 54] which is routinely used and repeated for colorectal cancer screening. Importantly, a bronchoscopy with brush biopsy limited to the *central airways* for collection of NBEC, the procedure used here, virtually eliminates risk for the primary complications reported to be associated with bronchoscopy, including pneumothorax or significant hemorrhage. Consistent with this, we observed no SAE associated with bronchoscopic brush biopsy in the subjects enrolled based on SD bronchoscopy.

It is particularly important to assess safety and comfort in the subjects meeting accepted criteria for lung cancer screening, a group that has increased prevalence for numerous comorbidities. Results from at least one previous report have suggested that research bronchoscopy and brush biopsy can be safely performed in subjects with heavy smoking history and those with obstructive lung disease [45]. Previous guidelines have suggested that an FEV1 less than 60 % is considered a contraindication to performing research driven bronchoscopy. However, bronchoscopy in adults with stable asthma and COPD has been performed safely at lower values of FEV1 [55]. Pulmonary function test data were available for more than 75 % of the subjects enrolled here (290 of 384 subjects). One hundred fifty-seven had clinical COPD and more than 70 % had GOLD stage 2 or worse (Table 2). Additionally, 9 % of enrolled subjects had a history of interstitial lung disease, 9 % were single-lung transplant recipients and a small percentage had other pulmonary disease (Additional file 3: Table S3) and bronchoscopy was safely performed on all of them. Specifically, no complications (SAEs) were associated with bronchoscopic brushing and sample collection in either standard of care (SOC) or study driven (SD) group.

In summary, bronchoscopic brush of the central airways to collect NBEC for lung cancer risk analysis was safe and well-tolerated in this study of subjects demographically at risk for lung cancer, including those with significant co-morbid conditions. Because the AE rate was much lower than that reported for routinely used screening colonoscopy [53] we expect that this procedure will be acceptable to patients and clinicians if the LCRT or other tests in development are validated to identify subjects with increased risk for lung cancer and/or early stage lung cancer.

### COPD characteristics of LCRT Cohort

The enrolled cohort had a high fraction of COPD based on PFT criteria. This is important because COPD is an independent risk factor for lung cancer [30–38]. Notably, using PFT data (FEV1/FVC <0.7) as the diagnostic criterion, one-third of individuals in this study misclassified their COPD status on the enrollment survey self-report. This is consistent with multiple reports of data acquisition through self-report leading to either misclassification or under-diagnosis of COPD [56–60]. Some of this misclassification could be due to patient being told they have COPD on the basis of radiographic imaging while the PFT data do not meet criteria for COPD diagnosis. Additionally, a portion of the subjects here underwent PFT at the time of enrollment that revealed COPD for the first time because the subject had not been tested prior to enrollment in the study. Given the importance of accurate COPD diagnosis, we plan to obtain both chest CT and PFT data from each subject at each subsequent follow-up. We will then evaluate COPD based on CT (presence of emphysema and/or bronchial thickening) or PFT criteria alone, or in combination as a risk factor for lung cancer.

### LCRT cohort and biospecimen repository as a resource for subsequent studies

As presented here, the LCRT cohort is well characterized with respect to demographic characteristics. In addition, NBEC and matching blood samples were collected from each subject. Each subject had a baseline CT scan and pulmonary function test (PFT) data are available for 76 % of individuals. It is planned to obtain a repeat PFT and CT scan on all subjects at each subsequent follow-up. This information will enable longitudinal assessment for rate of decline in pulmonary function by both physiologic and radiographic measures and to assess for presence or absence of lung cancer. More than 90 % of samples assessed so far passed QC quality and quantity criteria for reliable LCRT measurement. The NBEC and matching blood samples collected in this study are archived and the majority of subjects have given consent for use of samples remaining after LCRT analysis for future IRB approved studies. Currently, we are using NBEC gene expression data and genotyping data from matched peripheral blood cell gDNA to identify proximate phenotypic biomarkers for COPD risk and additional biomarkers for hereditary lung cancer risk. We are integrating these data with COPD genome wide association study (GWAS) data from the Lung Health Study and the COPDgene study available online at NCI dbGAP.

## Conclusions

The demographic composition of the enrolled group is representative of the population for which the LCRT is intended. Specifically, based on baseline population characteristics we expect lung cancer incidence in this cohort to be representative of the population eligible for LDCT lung cancer screening. Collection of NBEC by bronchial brush biopsy/bronchoscopy was safe and well-tolerated in this population. These findings support the feasibility of testing LCRT clinical utility in this prospective study. If validated, the LCRT has the potential to significantly narrow the population of individuals requiring annual low-dose helical CT screening for early detection of lung cancer and to enable safe delay the onset of screening for individuals with results indicating low lung cancer risk. For these individuals, the small risk incurred by undergoing once in a lifetime bronchoscopic sample collection for LCRT may be offset by a reduction in their CT-related risks. The LCRT biospecimen repository will enable additional studies of genetic basis for COPD and/or lung cancer risk.

## Additional files

Additional file 1:
**Lung Cancer Risk Test Study Enrollment by Study Site.** (DOCX 15 kb) Additional file 2:
**Work Type and Exposures.** (DOCX 15 kb) Additional file 3:
**Medical History.** (DOCX 15 kb) Additional file 4:
**Standard of Care (SOC) vs. Study Driven (SD) Bronchoscopies.** (DOCX 16 kb) Additional file 5:
**Self-reported vs. Clinical COPD.** (DOCX 14 kb) Additional file 6:
**Transplant vs Non-Transplant Subjects.** (DOCX 16 kb)

## Abbreviations

AE: Adverse event
COPD: Chronic obstructive pulmonary disease
FEV1: Forced expiratory volume in 1 second
FVC: Forced vital capacity
GWAS: Genome wide association study
LCRT: Lung Cancer Risk Test
LDCT: Low dose computed tomography
NBEC: Normal bronchial epithelial cells
PFT: Pulmonary function test
SAE: Serious adverse event
SNP: Single nucleotide polymorphism
SD: Study driven
SOC: Standard of care
USPSTF: United States Preventive Services Task Force
