# Supplementary material for: Lung cancer risk test trial: study design, participant baseline characteristics, bronchoscopy safety, and establishment of a biospecimen repository
Source: BMC Pulm Med. 2016 Jan 22;16:16. doi: 10.1186/s12890-016-0178-4 (PMC4722707; doi:10.1186/s12890-016-0178-4)
Supplement: Supplementary file 1 [file 12890_2016_178_MOESM1_ESM.docx]

Supplementary Table 1. Lung Cancer Risk Test Study Enrollment by Study Site

Site	Location	Subjects enrolled*
University of Toledo Medical Center	Toledo, OH	83
Mayo Clinic	Rochester, MN	43
University of Michigan	Ann Arbor, MI	86
The Toledo Hospital	Toledo, OH	19
Ohio State University	Columbus, OH	29
Vanderbilt University Medical Center/Tennessee Valley VA	Nashville, TN	25
Henry Ford Health System	Detroit , MI	6
National Jewish Health	Denver, CO	4
Medical University of South Carolina	Charleston, SC	20
Inova Fairfax Hospital	Falls Church, VA	14
Cleveland Clinic Foundation	Cleveland, OH	51
Mercy St. Vincent Medical Center	Toledo, OH	4
Total		384

* number in final cohort
