# Supplementary material for: Lung cancer risk test trial: study design, participant baseline characteristics, bronchoscopy safety, and establishment of a biospecimen repository
Source: BMC Pulm Med. 2016 Jan 22;16:16. doi: 10.1186/s12890-016-0178-4 (PMC4722707; doi:10.1186/s12890-016-0178-4)
Supplement: Supplementary file 2 [file 12890_2016_178_MOESM2_ESM.docx]

Supplementary Table 2. Work Types and Exposures

Total reported work exposures	n = 234/384 (61%)
Asbestos	54 (14%)
Baking	11 (3%)
Butchering/Meat Packing	13 (3%)
Chemicals/Plastics	39 (10%)
Coal Mining	4 (1%)
Cotton or Jute Processing	2 (<1%)
Farming	41 (11%)
Fire Fighting	8 (2%)
Flour, Feed or Grain Milling	7 (2%)
Foundry or Steel Milling	34 (9%)
Hard Rock Mining	1 (<1%)
Painting	33 (9%)
Sandblasting	15 (4%)
Welding	37 (10%)
