# Supplementary material for: Lung cancer risk test trial: study design, participant baseline characteristics, bronchoscopy safety, and establishment of a biospecimen repository
Source: BMC Pulm Med. 2016 Jan 22;16:16. doi: 10.1186/s12890-016-0178-4 (PMC4722707; doi:10.1186/s12890-016-0178-4)
Supplement: Supplementary file 3 [file 12890_2016_178_MOESM3_ESM.docx]

Supplementary Table 3. Medical History

Total enrollment	n = 384
Standard of care bronchoscopy	269 (70%)
Study driven (volunteer) bronchoscopy	115 (30%)
Standard of care CT scan	242 (63%)
Study driven CT scan	142 (37%)
Under investigation for lung cancer at enrollment	46(12%)
Family history of lung cancer	77 (20%)
Personal history of cancer	72 (19%)
Personal history of COPD (self-reported)	156 (41%)
Personal history of chronic bronchitis	68 (18%)
Personal history of emphysema	106 (28%)
Personal history of interstitial lung disease	35 (9%)
Personal history of sarcoidosis	10 (3%)
Personal history of scleroderma	1 (<1%)
Single lung transplant recipient	34 (9%)
