# Supplementary material for: Lung cancer risk test trial: study design, participant baseline characteristics, bronchoscopy safety, and establishment of a biospecimen repository
Source: BMC Pulm Med. 2016 Jan 22;16:16. doi: 10.1186/s12890-016-0178-4 (PMC4722707; doi:10.1186/s12890-016-0178-4)
Supplement: Supplementary file 4 [file 12890_2016_178_MOESM4_ESM.docx]

Supplementary Table 4. Standard of Care (SOC) vs. Study Driven (SD) Bronchoscopies

Baseline characteristics	SOC	SD	p value**
Total Enrolled	269	115	
Age in years [mean (SD*)]	63.6 (8.4)	61.5 (7.5)	p = 0.021
Male	153 (57%)	60 (52%)	
Female	116 (43%)	55 (48%)	
Caucasian	243 (91%)	100 (88%)	
African American	23 (9%)	14 (12%)	
Other or not reported	3 (1%)	1 (<1%)	
Cigarette pack years# [mean (SD)]	49.4 (24.2)	51.9 (22.8)	
Current smoker	67 (25%)	63 (55%)	
Former smoker	202 (75%)	52 (45%)	p < 0.001
COPD (all)$	120 (60%)	37 (41%)	p = 0.002
Stage 1	26 (13%)	19 (21%)	
Stage 2	62 (31%)	15 (16%)	
Stage 3	23 (12%)	2 (3%)	
Stage 4	7 (4%)	0 (0%)	

* SD = standard deviation

Pack years = packs of cigarettes smoked per day x years of smoking

$ staging info. unavailable for 2 subjects

** p value from Student's t-test reported if < 0.05
