# Supplementary material for: Lung cancer risk test trial: study design, participant baseline characteristics, bronchoscopy safety, and establishment of a biospecimen repository
Source: BMC Pulm Med. 2016 Jan 22;16:16. doi: 10.1186/s12890-016-0178-4 (PMC4722707; doi:10.1186/s12890-016-0178-4)
Supplement: Supplementary file 5 [file 12890_2016_178_MOESM5_ESM.docx]

Supplementary Table 5. Self-reported vs. Clinical COPD

PFT diagnostic criteria for COPD (all sites)

Self-reported	YES by PFT*	NO by PFT	No PFT data	
YES	92	32	32	
NO	58	94	51	
Did not self-report	7	7	11	
Accuracy				67%

* PFT = Pulmonary Function Test
