# Supplementary material for: Lung cancer risk test trial: study design, participant baseline characteristics, bronchoscopy safety, and establishment of a biospecimen repository
Source: BMC Pulm Med. 2016 Jan 22;16:16. doi: 10.1186/s12890-016-0178-4 (PMC4722707; doi:10.1186/s12890-016-0178-4)
Supplement: Supplementary file 6 [file 12890_2016_178_MOESM6_ESM.docx]

Supplementary Table 6. Transplant vs. Non-Transplant Subjects

Baseline characteristics	Transplant	Non-Transplant	p value**
Total Enrolled	34	350	
Age in years [mean (SD*)]	62.9 (8.5)	63.9 (3.7)	
Male	22 (65%)	191 (55%)	
Female	12 (35%)	159 (45%)	
Caucasian	31 (91%)	312 (89%)	
African American	3 (9%)	34 (10%)	
Other or not reported	0 (0%)	4 (1%)	
Cigarette pack years[#] [mean (SD)]	51.7 (27.2)	50 (23.5)	
Current smoker	0 (0%)	130 (37%)	
Former smoker	34 (100%)	220 (63%)	$p < 0.001$
COPD (all)[$]	21/34 (62%)	136/236 (53%)	
Personal history of interstitial lung disease	10 (29%)	25 (7%)	$p < 0.001$

* SD = standard deviation

** p value from Student's t-test reported if < 0.05

[#] Pack years = packs of cigarettes smoked per day x years of smoking

[$] in subjects for whom Pulmonary Function Test (PFT) data are available
